# Emergency contraception amongst female college students – knowledge, attitude and practice

**DOI:** 10.4102/phcfm.v6i1.538

**Published:** 2014-03-19

**Authors:** Wendwosen T. Nibabe, Tennyson Mgutshini

**Affiliations:** 1 Ethiopian Society of Obstetricians and Gynaecologists, Addis Ababa; 2 College of Human Sciences, University of South Africa, South Africa

## Abstract

**Background:**

Unwanted teenage pregnancies have a notable detrimental impact on the learners’ trajectory and have been associated with jeopardising the students’ educational progress and future career prospects. These pregnancies are mostly unplanned and unintended and many are terminated, either legally or illegally.

**Aim:**

The aim of this study was to explore the contributory role played by the knowledge, attitude and practices of female college students with respect to the utilisation of emergency contraceptives.

**Setting:**

Three tertiary institutions in Dessie, Ethiopia.

**Methods:**

Quantitative self-administered questionnaires were used to collect descriptive data from 352 female college students.

**Results:**

The study revealed that there was a high percentage (78.3%) of unwanted pregnancies amongst those engaging in sex. Significantly, nearly half (43.3%) of these unwanted pregnancies resulted in abortion. Only 10% of the students sampled admitted to ever having used emergency contraception. Even though more than half (69.9%) of the students knew about emergency contraception, only 27% of them felt confident that they understood when it was most effective.

**Conclusion:**

These and other observed findings confirm the need for improvement of female college students’ knowledge and timely utilisation of emergency contraception.

## Abstrait

**La contraception d'urgence chez les étudiantes inscrites dans l'enseignement supérieur – connaissances, attitudes et pratiques**

**Présentation:**

Les grossesses non désirées des adolescentes ont un impact négatif notoire sur les parcours des apprenantes et ont été associées à la mise en péril des progrès des étudiantes en termes d’études et de perspectives professionnelles. Ces grossesses sont, pour l'essentiel, non planifiées et non désirées, et nombre sont interrompues, que ce soit légalement ou illégalement.

**Objectif:**

L'objectif de l’étude était d’étudier le rôle joué par les connaissances, les attitudes et les pratiques des étudiantes inscrites dans l'enseignement supérieur quant à l'utilisation des contraceptifs d'urgence.

**Cadre:**

Trois établissements d'enseignement supérieur à Dessie, en Éthiopie.

**Méthodes:**

Des questionnaires quantitatifs auto-administrés ont été utilisés afin de recueillir des données descriptives auprès de 352 étudiantes suivant des études supérieures.

**Résultats:**

L’étude a révélé qu'il existait un fort pourcentage (78.3%) de grossesses non désirées parmi les jeunes filles prenant part à des activités sexuelles. Il convient de noter que près de la moitié (43.3%) de ces grossesses non désirées se soldait par un avortement. Seules 10% des étudiantes interrogées ont déclaré avoir utilisé une contraception d'urgence. Bien que plus de la moitié (69.9%) des étudiantes savait que la contraception d'urgence existait, 2% d'entre elles seulement étaient certaines de bien comprendre à quel moment celle-ci était la plus efficace.

**Conclusion:**

Ces conclusions, auxquelles viennent s'ajouter celles d'autres études, confirment la nécessité d'améliorer les connaissances des étudiantes inscrites dans l'enseignement supérieur sur la contraception d'urgence et son utilisation en temps opportun.

## Introduction

Emergency contraception is a method of preventing unwanted pregnancy resulting from unintended sexual activity, contraceptive failure or sexual assault,^1^ as well as from a lack of knowledge about or access to contraception. Its primary use is in reducing the need for abortions and the negative maternal health consequences associated therewith.^2^ Related literary sources suggest that, for most of the youth, tertiary education represents a shift toward greater independence from home – an opportunity to form new friendships and, for some, an opportunity to experience romantic and/or sexual relationships. Higher education students’ unwanted pregnancies pose a major public health problem globally. This problem also presents in Ethiopia, where there are added challenges resulting from the fact that some parts of society adhere to strict cultural and religious practices that forbid premarital sex. Unwanted pregnancies have a notable detrimental impact on the learners’ trajectory and have been associated with jeopardising the students’ educational progress and future career prospects. These pregnancies are mostly unplanned and unintended and many are terminated, either legally or illegally.^3^

Predecessor research suggests that ‘emergency contraception (EC) can substantially reduce a woman's chance of becoming pregnant when taken soon after sex’.^4–6^ By inference, this offers protection from the negative consequences of unplanned pregnancies as described above. When used within 72 hours after sexual contact, emergency contraceptive tablets have the capacity to prevent pregnancy by 75% - 58% and, when combined with the use of intra-uterine contraceptive devices (IUCDs), as many as 99% of unwanted pregnancies can be prevented.^7^ Although options for and information about emergency contraception have increased, further efforts are needed in order to improve women's access to this important backup method of birth control.^2^ In global terms, an estimated 46 million induced abortions are performed annually with 78 000 maternal deaths each year.^8^ In Ethiopia, unsafe abortions account for nearly 60% of all gynaecological admissions and almost 30.0% of all obstetric admissions.^9^ Approximately 22% – 54% of direct obstetric deaths are as a result of unsafe abortion. Emergency contraception is increasingly regarded as the primary means to reduce abortion rates.^10^ In Ethiopia, women who undergo induced abortions are more likely statistically to be below the age of 30 years and literate, often having studied beyond the level of secondary education.^11^

Some, including Ambaw,^12^ Tilahun,^3^ Mengist^13^ and Tamire and Enqueselassie,^11^ have conducted research in different universities in Ethiopia and all concur that the number of students who have a positive attitude toward sexual practice and the number of those who are experiencing penetrative sex and induced abortion are increasing. Despite this, their awareness with regard to preventing unintended pregnancy after incidental sex is relatively limited. It is this consensus that sets the backdrop for the acknowledgement that making emergency contraceptive pills (ECPs) accessible to college students is a crucial first step in helping to curb the growing rates of unintended pregnancy and unsafe abortion.^14^ Imbedded within this is the need for a clearer understanding of the factors that promote or limit students’ use of emergency contraceptives.

## Statement of the research problem

In spite of the high rate of unwanted pregnancies, the uptake of emergency contraception to prevent such an occurrence amongst college students at different colleges in Ethiopia is disproportionately low. The factors that influence utilisation patterns are as yet poorly understood. This poses a range of major public health problems – including an increased risk of complications associated with illegal abortions in the country – and may be associated with dropout and non-completion of education amongst students due to unintended pregnancies. This area of research has not been given much attention within the Ethiopian context and, to date, only a few non-research-based reports exist, with much of the discourse being conducted within mass media outlets. This background dictates a need for primary research to offer empirical insights into the reasons for low uptake of emergency contraception as a first step toward the reduction of pregnancy in college students. In keeping with this imperative, the current study looks specifically at the role by played by attitudes, knowledge and practice in determining the usage of emergency contraceptives. For the study, ‘attitude’ will be conceptualised as being a hypothetical construct that represent an individual's degree of like or dislike for something. This is often referred to as the attitude object. People can also be conflicted about or ambivalent toward an object, meaning that they possess both positive and negative attitudes simultaneously toward the item in question. Attitudes are judgements.^15^

## Objectives

In attempting to achieve the aim of the study, a number of key objectives were identified and include: (1) to assess the students’ pre-existing knowledge with regard to emergency contraception; (2) to assess students’ pre-existing attitudes about sex and how can these affect their use of emergency contraception; (3) to assess the utilisation level of emergency contraceptives amongst students; and (4) to utilise the findings to develop practical recommendations for service providers.

## Research method and design

### Study design and setting

There are six colleges in Dessie, Ethiopia, of which three were selected by a simple random sampling method. A quantitative descriptive cross-sectional study was conducted at these three colleges. Primary data were collected via self-administered structured questionnaires to assess female college students’ *Knowledge*, *Attitude* and *Practice* toward sex and emergency contraception.

### Study population and sampling

In this study, all female students in the selected colleges (*N* = 2554) were considered as a source population and the required sample size was drawn from this population. Multi-stage sampling was used to guide the selection process. To start, three colleges out of the total six colleges in the city were selected using simple random sampling. The selected colleges were Dessie Teacher's Education College (6 departments, 40 classes and 1110 female students), Admas University College (6 departments, 8 classes and 248 female students) and ALKAN University College (2 departments, 15 classes and 451 female students).

### Sample size calculation

The sample size was calculated using a one-sample population proportion formula. Assuming the proportion of students who are aware of emergency contraception to be 50% and assuming a non-response rate of 10%, the required sample was calculated using the following formula:

$$n=[(zá/{2)}^{2*}p(1-{p)}^{*}(D)]/d^{2}=[({1.96)}^{2}(0.5)(0.5)]/({0.05)}^{2}=384.16$$

Adding a 10% non-response rate, 384.16 +10% = 423 study subjects. But since the total population is less than 10 000 or *n/N* > 10% it was essential to use population correction. Therefore, nf = ni/[1 + ni/*N*] = 423/[1 + 423/2554] = 363.

Where:

*n* = the desired sample size

*p* = proportion of students who have knowledge about emergency contraceptives = 50% (used to obtain a maximum sample size as there was no previous study conducted in this regard in Dessie) (*zá*/2)^2^ = critical value at 95% confidence level of certainty (1.96)

d^2^ = the margin of error between the sample and the population = 5%

D = design effect (one stage)

nf = final sample size after correction

ni = initial sample size before correction

The required sample size was thus 363.

Based on proportional allocation, 223 students from Dessie Teacher's Education College, 50 students from Admas University College and 90 students from ALKAN University College were included in the study.

### Data collection

Data were collected using a structured self-administered questionnaire. To assess whether the instrument covered all dimensions of the construct, relevant literature and experts in the field were consulted, with due consideration to the research problem. In addition, the instrument items were adapted in part from a standardised questionnaire used in the nationwide survey conducted by the Ethiopian Family Guidance Association and others from instruments of similar studies conducted and published in different journals.^16^ The questionnaires were pre-tested with students in two departments at Dessie Teacher's Education College. Based on pre-test feedback and suggestions, some questions were rephrased or amended and the final questionnaire was then prepared. Of the total questionnaires distributed to the respondents, five were incomplete and six were returned blank, resulting in a response rate of 97%.

### Data collection process

The questionnaire was administered at the three colleges over a number of days. There were three data collectors in total with each collecting data from one college. Randomly-selected students from different departments and different classes were divided into groups of 10 and distributed into different groups.

The students were briefed about the purpose of the initial meeting and very detailed information about the study was presented when they gathered in the lecture halls. They were then informed about the survey – its objectives and procedures – and were assured that the information collected would be treated as confidential and used only for research purposes. This approach was chosen so as to aid the dissemination of information in the most convenient manner. The interactive presentation of the study overview allowed potential participants opportunities to seek clarification. The purpose of this was to reduce the non-response rate. After these initial information sessions, prospective respondents were given a week-long cooling-off period to consider their decision about giving consent. In addition to this, the students were reassured throughout all stages of the study about their right to opt out of the study without repercussions. Students who gave consent were provided with the questionnaire and were given access to an area where they could complete their questionnaire in private.

### Validity and reliability

To promote validity and reliability within the study, a number of measures were introduced, including ensuring homogeneity in the ages of the students surveyed. For example, the study focused primarily on students at the same level of study. The students also had to meet pre-identified criteria, such as being 18 years or older and being a fulltime student, which helped to eliminate any bias resulting from differential selection of study subjects. There were, however, elements within the study which introduced some heterogeneity, for example, the participants were from different cultures and religions and included members from both rural and urban areas.

### Data management and analysis

A total of 352 female college students at the three chosen institutions completed the questionnaire. The data from the questionnaires were analysed by a statistician using SPSS for Windows, version 16.0. Descriptive statistics were elicited for each of the questions.

### Ethical issues

Multi-site ethical clearance was obtained from the higher degrees committee of the Department of Health Studies at the University of South Africa as well as from each of the three colleges that were included in the study. Written permission was provided by each of these entities with the express proviso that participating students would be guaranteed the freedom to refuse participation without fear of prejudice. In keeping with this, prospective participants were invited to take part in the study by letter initially and this was then followed up verbally on their arrival at the research site. Privacy and confidentiality were maintained both during and after the administration of the questionnaire by ensuring that the students in the lecture halls were separated out adequately and by posting individual questionnaires using registered post. At the beginning and at the completion of each questionnaire, data collectors thanked the study subjects for their willingness and for giving their time to participate in the study.

## Results

### Demographic data

All respondents were aged between 18 and 25 years. Of this group, 29.8% (*n* = 105) participants were between 18 and 19 years old; 34.1% (*n* = 120) were between 20 and 21 years old; 18.5% (*n* = 65) were between 22 and 23 years old; and 17.6% (*n* = 62) were between the ages of 24 and 25. For the religious breakdown of the respondents, 67.3% (*n* = 237) were Orthodox Christians; 30.1% (*n* = 106) were Muslim; 2% (*n* = 7) were Protestant; 0.3% (*n* = 1) were Catholic; and 0.3% (*n* = 1) had other affiliations. The educational level of the respondents ranged from first year (25%; *n* = 88) to third year (40.62%; *n* = 143), as indicated in Table 1.

**TABLE 1:** Age distribution of female college students.

Age of respondents in years	Frequency	Percent	Valid Percent	Cumulative Percent
18–19	105	29.8	29.8	29.8
20–21	120	34.1	34.1	63.9
22–23	65	18.5	18.5	82.4
24–25	62	17.6	17.6	36.9

**Total**	**352**	**100.0**	**100.0**	**100.0**

Of the respondents, 83.24% (*n* = 293) were unmarried and 16.76% (*n* = 59) married. The majority of the respondents (37.5%; *n* = 132) lived with their friends; 34.1% (*n* = 120) lived alone; and 28.4% (*n* = 100) lived with their family.

Identified independent variables were fit into five separate binary logistic-regression models (models 1–5) to examine the association between each of the predictor and outcome variables (emergency contraception awareness; attitude toward sex and emergency contraception; and sexual practice and utilisation of emergency contraception). In model 1, the dependent variable was dichotomised and coded as 1 (aware of emergency contraception) and as 0 (never heard about emergency contraception). In a similar fashion, the dependent variables (attitude toward sex and attitude toward emergency contraception) in model 2 and 3 were dichotomised and coded as 1 (favourable attitude) and 0 (unfavourable attitude). The fourth and fifth independent variables were dichotomised and coded as 1 (ever practised) and 0 (never practised). Categorical independent variables were categorised meaningfully and then grouped.

### Students’ knowledge of emergency contraceptives

One hundred and eighty-seven (53.3%) of the students knew about at least one regular modern contraceptive method and 79 (22.4%) knew about two or more modern methods, which is lower than a similar study conducted in the Oromia regional state of Ethiopia (85.6%).^13^ Amongst the modern methods known of by the respondents, injectable contraceptives were the most commonly-known method (22.7%), followed by pills (12.5%). On the other hand, 22.7% (*n* = 80) of the students did not know about any of the commonly-used contraceptive methods.

Of the 352 respondents, 69.9% (*n* = 246) had heard about emergency contraception. Of those who knew about emergency contraception, 8.9% (*n* = 22) had obtained their information from reading leaflets, 38.6% (*n* = 95) from radio and TV, 30.9% (*n* = 76) from health education at different health facilities and 21.5% (*n* = 53) from college during different lectures and peer discussion. Regarding the type of contraceptives used as emergency, 53.7% (*n* = 132) said pills, 11.4% (*n* = 28) said IUCD and 19.1% (*n* = 47) cited both pills and IUCD. Of these students, 15.9% (*n* = 39) did not know which particular contraceptive is used as emergency contraception. With respect to the type of drug used as emergency contraception, 46.3% (*n* = 114) replied that it is the same as the drug found in ordinary contraceptives and 41.9% (*n* = 103) answered that it is the same one, but stronger than the ordinary contraceptives. Concerning the time limit for taking pills and the insertion of an IUCD as emergency contraception, only 32.1% (*n* = 79) indicated correctly that emergency contraceptive pills should be taken within 72 hours, whilst only 6.5% (*n* = 16) replied that an IUCD should be inserted within 5 days.

To assess the level of actual knowledge, a series of eight knowledge questions (on method identification; drug composition; time frame for effective use; time interval between doses; effectiveness of the drug; appropriate situations for use; and places where emergency contraception can be found) were posed to those students who had heard of emergency contraception. To generate the summarised level of knowledge, the response on each question was first scored and tallied and then the total of each respondent was scored ranging from 0–8 (0% – 100%). A cumulative/total score was calculated and then the respondents were classified as poor, fair or good with respect to their level of knowledge about emergency contraception. Hence, respondents who scored zero were considered as ‘*not having the knowledge’*; those who scored 12.5% – 50% as ‘*fair knowledge’*; and those who scored more than 50% as ‘*good knowledge’*.

### Attitude of students toward sex

Students were questioned extensively about their attitudes toward sexual relationships. A set of nine statements pertaining to relationships and sex were included in the questionnaire. Six positive and three negative items were included in order to maintain the balance of responses. The nine items were answered as either ‘agreed strongly’, ‘agreed slightly’, ‘had no opinion about the statement under consideration’, ‘disagreed slightly’, or ‘disagreed strongly’ (a five-point Likert scale). For positively-worded statements, those who selected ‘agree’ were regarded as having a positive attitude and those who chose ‘disagree’ were considered to have a negative attitude. Conversely, for negatively-worded statements, those who selected ‘agreed’ were clustered as having a negative outlook, whereas those who said ‘disagree’ were categorised as having a positive attitude. The responses on each attitudinal items were scored and tallied, then the total of each respondent's score was ranged from 0–9 (0% – 100%). A score of 50% and above was considered to be a ‘*favourable attitude’* whereas those scoring below 50% were thought of as having an ‘*unfavourable attitude’*. The summarised attitudinal index indicates that 84.4% of the total respondents had a favourable attitude toward sexual relationships.

### Attitude toward emergency contraceptives

Respondents were again questioned about their attitudes toward emergency contraception. The five items were answered as either ‘agree strongly’, ‘agree slightly’, ‘neutral’, ‘disagree slightly’ or ‘disagree strongly’. For positively-worded statements, those who selected ‘agree’ were regarded as having a positive attitude and those who chose ‘disagree’ were considered to have a negative attitude. Conversely, for negatively-worded statements, those who selected ‘disagree’ were clustered as having a positive outlook whereas those who said ‘agree’ were categorised as having a negative attitude. The responses on each attitudinal item were scored and tallied, then the total of each respondent's score was ranged between 0 and 5 (0% – 100%). A score of 50% and above was considered as being a ‘*favourable attitude’*, whereas those scoring below 50% were thought of as having an ‘*unfavourable attitude’*.

### Respondents’ sexual practice

Respondents were asked whether they had ever had sexual intercourse and, if so, whether they had a history of pregnancy and at what age they became pregnant. Amongst the respondents, 36.6% (*n* = 129) had experienced sexual intercourse. Of the total 129 students that had sexual experience, 46.5% (*n* = 60) had a history of pregnancy. The majority (76.7%; *n* = 45) of these students had fallen pregnant between 15 and 19 years of age. Of these pregnancies, 78.3% (*n* = 47) were not planned. Of the total pregnancies, 43.3% (*n* = 26) resulted in an unsafe abortion.

### Respondents’ utilisation of emergency contraception

Of the 246 students who had heard about emergency contraception, only 15.4% (*n* = 38) of the students made use of it, which is still higher than a study conducted in Adama University and Jimma University of Ethiopia (4.7% and 6.8%, respectively).^3, 10^ The higher use of emergency contraception in this study could be because of the fact that a higher proportion of them (36.6%; *n* = 129) had a history of sexual intercourse when compared with those at Adama University3 (29%; *n* = 194).

With regard to regularity of use, 71.1% (*n* = 27) of the students had used emergency contraception only once and 28.9% (*n* = 11) of them had used it twice. Of the students who had ever used emergency contraception, 68.4% (*n* = 26) obtained it from nurses and the rest (31.6%; *n* = 12) directly from pharmacists. The majority of them (63.2%; *n* = 24) used it because of miscalculation of their safe sexual time. Most of the sexually-active respondents who had a history of sexual practice (56.6%; *n* = 73) complained that the main challenge with regard to utilisation of emergency contraceptives was their unavailability in pharmacies at the time of need. The second challenge mentioned by 23.3% (*n* = 30) of the respondents was fear of stigma. A diagrammatic overview is presented in Table 2.

**TABLE 2:** Practice with regard to emergency contraception amongst female college students at Dessie Teacher's Education College, ALKAN University College and Admas University College in Dessie, Ethiopia (2012).

Practice indicators	Number	Percent
**Have you ever used emergency contraceptive pills? (** ***n*** **= 246)**		
Yes	38	15.4
No/Others	208	84.6
**How many times have you used this method during the last year? (** ***n*** **= 38)**		
Once	27	71.1
Twice/Others	11	28.9
**Who recommend use of emergency contraception? (** ***n*** **= 38)**		
A friend	4	10.5
Partner (male)	9	23.7
Health professional/Others	25	65.8
**Who provided you with emergency contraception? (** ***n*** **= 38)**		
Nurses	26	68.4
Pharmacists/	12	31.6
**Why did you use emergency contraception? (** ***n*** **= 38)**		
Time was miscalculated	24	63.2
Condom broke	3	7.9
Pills missed	6	15.8
Withdrawal failed/Others	5	13.2
**What were the challenges you faced to get emergency contraception? (** ***n*** **= 129)**		
Price	6	4.7
Not available in pharmacies	73	56.6
Fear of stigma	30	23.3
Lack of knowledge/Others	20	15.5

## Discussion

### Knowledge of students about emergency contraceptives

Similar procedures were followed by Ayana^16^ and Desta and Regassa.^17^ Based on the summary index, about 66.1% fell into the range of ‘fair knowledge’ and only 33.9% had good knowledge of emergency contraception. The level of knowledge here is comparable to that conducted in Bahirdar University (34.8%)^18^ and slightly higher than a study conducted in Jimma University (22.8%),^19^ but lower than a study conducted in Addis Ababa University (43.5%).^11^

Binary logistic-regression analysis showed that students aged 18–19 and 20–21 are respectively 86.5% and 74.5% less knowledgeable than their reference age (24–25), with *p* < 0.001, adjusted odds ratio (AOR) 0.135, 95% confidence interval (CI) 0.056–0.323 for the students aged 18–19; and *p* < 0.005, AOR 0.255, 95% CI 0.106–0.610 for students aged 20–21. In the case of level of education, as years of study increased there was a relative increase in the knowledge about emergency contraception, making third-year students a reference (*p* < 0.001, AOR = 0.077, (95% CI 0.035–0.170) for first-year students and for second-year students (*p* < 0.001, AOR 0.141, 95% CI 0.066–0.301), which means they were respectively 92% and 86% less knowledgeable than third-year students. There is also a statistically-significant association between marital status and knowledge about emergency contraception. Married respondents had more knowledge than unmarried respondents (AOR 2.094, 95%CI 1.040–4.213).

### Attitude of students toward sex

In model 2, level of education had a significant association with attitude toward sex, where those in first year were 0.258 times less likely to have a favourable attitude toward sex than respondents at the third-year level.

### Attitude toward emergency contraceptives

A similar procedure was followed by previous researchers.^18, 20^ The summarised attitudinal index indicates that 63.6% of the respondents who had ever heard of emergency contraception had a favourable attitude toward it. This figure is better than studies conducted in Addis Ababa University (53%)^11^, Bahirdar University (56.7%)^18^ and Hawassa post-secondary school female students (65.6%),^16^ but less than a study conducted amongst female students at Haramaya University.^17^

In this study, there is also a statistically-significant association between age and educational level of the respondents and their attitude toward emergency contraception. Students aged 18–19 showed 86.5% less-positive attitudes towards emergency contraception (*p* < 0.001, 95% CI 0.056–0.323) compared with students aged 24–25. Similarly, students aged 20–21 were 74.5% less positive toward emergency contraception compared with their 24–25 year-old counterparts (*p* < 0.005, 95% CI.0.106–0.610). Again, first- and second-year students reported less-positive attitudes toward emergency contraception than third-year students (*p* < 0.001, AOR 0.077, 95% CI 0.035–0.170; and *p* < 0.0.001, AOR 0.141, 95% CI 0.066–0.301, respectively).

### Respondents’ sexual practice

There are no independent variables having a statistically-significant association with model 4 (sexual practice), except the marital status of the respondents – unmarried students are less likely to indulge in sexual practice (*p* < 0.001, AOR 0.0.370 and 95% CI 0.124–0.535). A similar study in Addis Ababa showed a lower rate of unwanted pregnancy (73.5%; *n* = 36),^2^ an equivalent rate of induced abortion (71.7%; *n* = 38) and a lower rate of unsafe abortion (29%; *n* = 11). The possible explanation for the low rate of safe abortion, high rate of unsafe abortion and high rate of unwanted pregnancy and delivery in this study could be due to a lack of health facilities with sufficiently-trained staff; a lack of awareness about where to get safe preventive methods; and financial problems that made the respondents take measures that threatened their life or their future.

### Respondents’ utilisation of emergency contraception

Of the variables that were studied, only two independent variables emerged as being statistically significant in terms of their association with utilisation of emergency contraception, namely, (1) age of the respondents and (2) marital status. Students aged from 22–23 years old were 2.9 times more likely to utilise emergency contraception than younger fellow students (*p* < 0.05, 95% CI 1.007–8.368) and married students were 0.359 times less likely to utilise emergency contraception than unmarried students (*p* < 0.005, 95% CI 0.169–0.760).

## Limitations of the study

There were a number of limitations with regard to this study. Firstly, the data were collected only from female students in colleges. In the rural areas, where only a small proportion of individuals get an opportunity to attend college, the result from the current study has limited statistical power and cannot be generalised to all youth in the study area, thus possibly overestimating the result. Secondly, self-reported information is subjected to reporting errors, missed values and bias. Since the study touches on sensitive issues, the possibility of underestimation cannot be excluded, even though the study was anonymous. Thirdly, the young females were extremely impatient during the collection, which was expected for this age. They were impatient with regard to the questions presented, as well as the time required. Many felt shy with respect to some issues, especially those related to sex, and there were 11 non-responses. Lastly, the current study offers a quantitative exploration and future researchers could offer clarifying insights through the use of qualitative methodologies.

## Conclusion

The study findings revealed that whilst overall awareness of emergency contraception was within the expected norms, specific knowledge of is very low (33.9%) amongst the female students of Dessie Teacher's Education College, Admas University College and ALKAN University College. Of those who had ever heard of emergency contraception, 63.6% reported favourable attitudes toward emergency contraception. The present study has also documented that the knowledge and attitude of female college students toward emergency contraception was affected by a range of personal characteristics including age and level of education, whilst marital status, attitude towards sex and utilisation of emergency contraception are affected by level of education and marital status. Finally, a high rate of sexual activity and unwanted pregnancies have been reported. This prompts a need for sexual health services to initiate important measures, such as provision of continuous sex education, guidance and counselling services – especially during the first year of university – and increasing accessibility of emergency contraception and other preventive methods to the users. It is worth noting that the campus health workers can play an important role by spreading the knowledge of emergency contraception widely throughout the student community through individual counselling when female students visit the clinic.

### Recommendations

Strengthening information education and communication in colleges on sexual reproductive health, with special emphasis on different family-planning methods – including emergency contraception – will be a problem-solving procedure for female college students. Contraception information sessions should cover all details of how the emergency contraceptives work and how they should be taken.

Clinics guided by special policies should be available to provide reproductive health services for adolescence and young female students, in particular over weekends and during the evenings.

A collaborative effort between service providers, health institutes and colleges is of paramount importance. It would be a good idea to make emergency contraceptives, especially emergency contraceptive pills, available at all drug-dispensing institutes including private and government institutions, non-governmental organisations, pharmacies, clinics and community-based distribution agents. Facilitating conditions to distribute or sell without prescription, after sufficient training and information sessions for both dispensers and clients, could be helpful.

There is also a need to empower young people to discuss sexual and reproductive health issues with their parents, friends and others.

The majority of the female college students did not have any actual knowledge about emergency contraceptives. It is thus recommended that emergency contraceptives be advertised in clinics, in colleges, at schools and also during radio and television broadcasts.

Strengthening of sexual health clubs starting from the lower levels of education is also important. Educators should provide moments to reflect, to think about the responsibilities involved in sexual practices and to discuss gender relations in terms of relationships because this aspect is linked directly to pregnancy in female college students. Emergency contraception should also be included in the curriculum of the students along with other family planning methods.
